# The Variability of Thymol and Carvacrol Contents Reveals the Level of Antibacterial Activity of the Essential Oils from Different Accessions of *Oliveria decumbens*

**DOI:** 10.3390/antibiotics9070409

**Published:** 2020-07-14

**Authors:** Tahereh Khoshbakht, Akbar Karami, Aminallah Tahmasebi, Filippo Maggi

**Affiliations:** 1 Department of Horticultural Science, School of Agriculture, Shiraz University, 71441-65186 Shiraz, Iran; t.khoshbakht93@gmail.com (T.K.); akbarkarami@shirazu.ac.ir (A.K.); 2 Department of Agriculture, Minab Higher Education Center, University of Hormozgan, 84156-83111 Bandar Abbas, Iran; Tahmasebi.info@yahoo.com; 3 School of Pharmacy, University of Camerino, 62032 Camerino, Italy

**Keywords:** *Oliveria decumbens*, essential oil, antibacterial activity, chemical composition, populations

## Abstract

*Oliveria decumbens* (Apiaceae) is an aromatic herb traditionally employed in the Persian medicine for the treatment of infectious and gastrointestinal disorders. In the present study, we analyzed the chemical composition of essential oils obtained from different Iranian populations and evaluated their efficacy on a panel of human pathogens (*Staphylococcus aureus* and *Escherichia coli*), probiotic (*Bacillus subtilis*), and phytopathogens (*Clavibacter michiganensis*, *Curtobacterium flaccumfaciens*, *Xanthomonas citri*, and *Agrobacterium tumefaciens*). The gas chromatographic-mass spectrometry analysis put in evidence four main volatile constituents such as thymol (20.3–36.4%), carvacrol (18.8–33.1%), γ-terpinene (10.6–25.9%), and *p*-cymene (9.5–17.3%), though with significant variability from an essential oil to another. Notably, the oils from the populations sited in Nourabad Mamasani and Dehdasht showed the highest amount of the phenolic monoterpenes thymol (36.4 and 35.2%, respectively) and carvacrol (33.1 and 30.6%, respectively). The antibacterial activity of *O. decumbens* essential oils was assessed by the minimum inhibitory concentration (MIC) and minimum bactericidal concentration (MBC) methods, showing high activity for the samples from Nourabad Mamasani and Dehdasht populations exhibiting high level of the above phenolics. The obtained MIC and MBC values (mg/ml) were in the ranges 0.0625–2 mg/ml and 1–16 mg/ml, respectively. Noteworthy, in some cases, the antibacterial activity of *O. decumbens* essential oils was higher than that of chloramphenicol used as positive control. The average MBCs displayed by the *O. decumbens* samples showed that *C. flaccumfaciens* had the highest sensitivity to the essential oils. Based on these results, our work shed light on selected *O. decumbens* populations deserving proper breeding and cultivation strategies in order to warrantee production of bioactive essential oils to be used at pharmaceutical and agricultural level to combat several pathogens.

## 1. Introduction

*Oliveria decumbens* Vent., belonging to the Apiaceae family, is an annual, aromatic herb, endemic to southern and western parts of Iran [1]. The aerial parts of this plant have been used to cure fever, abdominal pain, indigestion, and diarrhea [2]. Moreover, *O. decumbens* is exploited in Persian traditional medicine for the treatment of cancer, infections, and inflammation [3,4,5]. *Oliveria decumbens* is a good source of essential oil which has already been reported to exhibit notable antioxidant activity [6]. Notably, an *O. decumbens* emulsion exhibited strong radical scavenging effects and suppressed lipid peroxidation in both chemical assays and living cells [7]. This essential oil has also been reported to have insecticidal activity against the cabbage looper *Trichoplusia ni* (Hübner) [8]. Finally, the *O. decumbens* essential oil showed cytotoxic effects on several cancer cell lines [8,9]. Actually, essential oils are natural sources of volatile, lipophilic mixtures of several dozens of compounds which are able to interact with the cell wall, increasing its permeability, or disrupting the energy production system of the cells. In addition, they exert an important inhibition of trans-membrane and cytosol enzymes as well as anti-quorum sensing activity [10,11,12]. 

In this regard, the antibacterial and antifungal activities of *O. decumbens* essential oils have been disclosed in previous papers [8,13,14,15,16,17,18]. *Staphylococcus aureus* and *Escherichia coli* are opportunistic pathogens that cause infections in humans and the previous studies have emphasized the development of resistance in these bacteria to antibiotics [19]. *Bacillus subtilis* is considered as a probiotic bacterium with the beneficial effects in the animals and plants [20]. Plant pathogenic bacteria cause major constraints and losses on crop yield worldwide [21]. *Clavibacter michiganensis* as an economically important pathogen causes severe losses and destructive diseases in agricultural crops [22]. Also, *Curtobacterium flaccumfaciens* causes important emerging disease and serious problems in the legumes around the world [23]. Moreover, *Xanthomonas citri* is an important and serious pathogen causing citrus canker in citrus plants [24]. *Agrobacterium tumefaciens* is regarded as a serious and widespread plant pathogenic bacterium causing crown gall disease [25].

Considering the development of bacterial resistance to chemical antibiotics and their adverse effects on the environment and public health, there is a growing demand to replace them with plant-based derivatives or combine these green agents with antibiotics to give synergistic effects [26,27,28,29]. In this respect, plant essential oils have shown efficacy on many bacterial and fungal strains, without emergence of noteworthy resistance [28]. Thus, essential oils are promising antimicrobial sources to be employed in the years to come as an effective alternative to combat multi-drug resistant pathogens [26,27,28,29]. 

The chemical composition of the essential oils is affected by various factors including environment, climate, genetics, harvesting time, extraction methods, and others [30,31,32]. Previous studies revealed that the essential oil composition and content in *O. decumbens* collected from different regions of Iran varied significantly [2,7,30,33,34,35,36]. 

The main constituents and their possible synergistic interactions determine the biological activities of plant essential oils [37]. Thus, the variation in chemical composition detected among various populations may affect the level of biological properties of a plant species, compromising in some cases its use on an industrial level. A previous study showed that essential oils from five populations of *O. decumbens* collected in different regions showed no significant differences in antimicrobial activity [17]. With regard to possible differences in chemical composition among different *O. decumbens* accessions [2,7,30,33,34,35,36], it is of industrial importance to screen and select those populations characterized by the best fingerprint, in terms of bioactive compounds assuring a strong and reliable antibacterial activity. Therefore, the present study was aimed to investigate the chemical variability of essential oils in *O. decumbens* growing in different Iranian regions and to evaluate its effect on the antibacterial activity exerted on different bacterial strains of human pathogens and phytopathogens. 

## 2. Results and Discussion

### 2.1. Essential Oil Content

The essential oil content differed among the various *O. decumbens* populations and ranged from 3.34 to 8.52% (w/w) [33]. On the other hand, the values reported in previous reports varied from 0.1 to 8.1% [2,15,16,18,30,38,39]. The highest and lowest contents were found in Behbahan and Nourabad Mamasani populations with 8.52 and 3.34%, respectively. Moreover, Dehdasht (5.19%) and Kahnoyeh (5.32%) populations showed no significant differences. The essential oil content of Behbahan population, with 8.52%, showed a remarkable difference compared to those previously reported for several *O. decumbens* populations [2,15,18,30,38,39]. A number of various factors, including genetics, soil, geographic, and climatic conditions affect the essential oil content [40]. In addition, some factors like altitude have been reported to affect the essential oil content [33,40,41]. In our study, the highest content was found in the essential oil from Behbahan population growing at the lowest altitude (394 m), followed by that from Kahnoyeh and Dehdasht populations located at 758 and 971 m a.s.l., respectively. Also, the oil from Nourabad Mamasani population, growing at the highest altitude (1268 m), showed the lowest content. Based on our results, we can conclude that the highest essential oil contents were obtained at lower altitudes. This finding is in accordance with that reported in another study in which the authors showed a significant negative relationship between the *O. decumbens* essential oil content and the altitude [41]. Thus, fields at low cultivation altitudes should be considered to produce essential oils with high yield. 

### 2.2. Essential Oil Composition

Our recent study showed that the number and relative percentages of essential oil compounds changed at variance with the geographic origin of *O. decumbens* populations [33]. A total of 18, 17, 16, and 11 compounds were identified in the populations from Nourabad Mamasani, Kahnoyeh, Behbahan, and Dehdasht, respectively [33]. The number of identified compounds in the investigated populations was partly in accordance with those reported in previous papers, that ranged from 8 to 35 [2,7,9,14,15,16,17,18,30,38]. 

The main essential oil constituents were thymol (20.3–36.4%), carvacrol (18.8–33.1%), γ-terpinene (10.6–25.9%), and *p*-cymene (9.5–17.3%). Quantitatively they showed a significant variability at variance with the geographic origin of the plant material. The highest amount of thymol was found in Nourabad Mamasani and Dehdasht populations, with 36.4 and 35.2%, respectively, whereas the lowest one (20.3%) was found in the Kahnoyeh population. Moreover, the highest percentage of carvacrol was found in the Nourabad Mamasani and Dehdasht populations, with 33.1 and 30.6%, respectively, while the lowest one was detected in the Kahnoyeh population (18.8%). γ-Terpinene, which is related to the biosynthesis of the phenolic monoterpenes [42], showed an inverse trend, with the highest percentage (25.9%) in the essential oil from the Kahnoyeh population, and the lowest one in that from Nourabad Mamasani (13%). A similar trend was observed for *p*-cymene, with the oil from Behbahan population containing the highest percentage (17.3%), and that from Nourabad Mamasani showing the lowest value (9.5%) [33]. 

The essential oil chemical profiles of *O. decumbens* [33] detected in this study were in agreement with those described in previous studies in which thymol, carvacrol, γ-terpinene, and *p*-cymene resulted the major volatile constituents [2,7,30,36,38]. The variation in the number and content of essential oil constituents may be ascribable to factors including genetics, climatic, and geographic conditions, chemotype, collection time, and extraction technique [30,31,40]. It has been shown that geographical and meteorological factors affect the content and composition of *O. decumbens* essential oil [33,41]. Indeed, the essential oil content in *O. decumbens* showed a significant positive correlation with the soil sand content and temperature, while it revealed a negative correlation with the altitude [33]. In our study, the *O. decumbens* populations were collected at the same stage and time (flowering stage, 1–21 June 2017), thus we can exclude the effect of the phenological cycle. Also, the extraction and analytical methods were conducted in the same way. Therefore, we assume that the climate, environmental, and geographic conditions play a major role in affecting the essential oil chemical composition. 

### 2.3. Antibacterial Activity

The minimum inhibitory concentration (MIC) and minimum bactericidal concentration (MBC) values (mg/ml) of the *O. decumbens* essential oils were in the ranges 0.0625–2 mg/ml and 1–16 mg/ml, respectively (Table 1 and Table 2). The mean of the MICs and MBCs showed that the Gram-positive bacteria had a higher sensitivity than the Gram-negative bacteria. Therefore, our findings are in accordance with previous studies showing a major impact on the Gram-positive bacteria [15,17,43]. The differences in antibacterial rate displayed by the *O. decumbens* essential oils may be attributed to the variation in the structure of cell wall in Gram-positive and negative bacteria as the cell wall of the latter is surrounded by an external membrane [10,14].

Moreover, the essentials oil from Nourabad Mamasani and Dehdasht populations showed the best performance in terms of MIC and MBC values, highlighting the highest inhibitory activity against both groups of bacteria. On the other hand, the essential oil from the Kahnoyeh population showed the lowest antibacterial activity with the highest MIC and MBC values. The strongest antibacterial activity of the essential oils from Nourabad Mamasani and Dehdasht populations might be attributed to the highest content of thymol (36.4 and 35.2%, respectively) and carvacrol (33.1 and 30.6%, respectively) and their possible synergism [44]. On the other hand, the oil from the Kahnoyeh population, with the lowest amount of thymol (20.3%) and carvacrol (18.8%), showed the weakest inhibitory activity. These differences in the content of thymol and carvacrol as the most important antimicrobial compounds, may explain the variability and level of the antibacterial activity among the four *O. decumbens* essential oils as a positive correlation exists between the content of thymol and carvacrol and the antibacterial efficacy [45]. As a matter of fact, Pearson correlation analysis was conducted to evaluate the relationship between the thymol and carvacrol contents and the antibacterial activity (MICs and MBCs). Results showed that thymol had a significant positive correlation with the carvacrol content (r = 0.996, significant at the 0.01 level), thus the thymol content increased together with that of carvacrol. Taken together, the thymol and carvacrol contents showed a significant negative correlation with MIC values (r = 0.984 and 0.979, respectively, significant at the 0.01 level) meaning that the most active essential oils were the richest in these compounds. In addition, the MBC values exhibited a non-significant positive correlation with MIC values (r = 0.729) and a non-significant negative correlation with the thymol and carvacrol contents (r = 0.833 and 0.851). 

**Figure 1:**
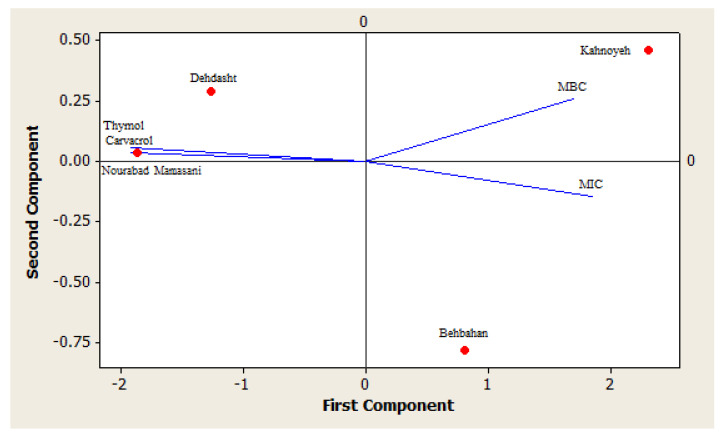
Biplot of the first two principal components (PCs) for the thymol and carvacrol contents and the MIC and MBC values of *Oliveria decumbens* populations.

The thymol and carvacrol contents and the MIC and MBC values were selected for principal component analysis (PCA). The results of PCA based on PCI and PCII showed that Nourabad Mamasani and Dehdasht populations were placed into the same group, while Behbahan and Kahnoyeh populations belonged to two distinct groups (Figure 1). The essential oils from Nourabad Mamasani and Dehdasht populations were placed closer to the lines of thymol and carvacrol contents highlighting the greatest correlation with these components. On the other hand, the oil from Kahnoyeh population was placed close to MBC showing the lowest bactericidal activity (Figure 1).

Our results are in agreement with previous studies which documented the effectiveness of thymol and carvacrol compounds against various bacteria [46]. In addition, thymol and carvacrol were suggested as the bioactive compounds against *Escherichia coli*, *Staphylococcus aureus,* and *Candida albicans* [17]. Thymol and carvacrol inhibit the bacterial growth and ergosterol biosynthesis and also disrupt the bacterial membrane, resulting in ions and ATP leakage, and cell death [14,47,48]. The average MICs and MBCs displayed by the essential oils indicated that *Clavibacter michiganensis* and *Agrobacterium tumefaciens* were the most susceptible phytopathogens with the lowest MIC values (0.44 mg/ml). While, *Xanthomonas citri*, with the highest MIC values, showed the strongest resistance to the essential oils. In some cases, the essential oils activity was better than that of chloramphenicol used as positive control (Table 2 and Table 3). Indeed, the essential oils from Nourabad Mamasani, Dehdasht, and Behbahan populations inhibited *Bacillus subtilis*, with MIC values (0.0625, 0.25, and 0.5 mg/ml, respectively) lower than that of chloramphenicol. Moreover, the oil from Nourabad Mamasani showed the strongest activity against *E. coli*, with a MIC value (0.25 mg/ml) lower than that of chloramphenicol (Table 2). Furthermore, considering the average MBC of all essential oils assayed, *Curtobacterium flaccumfaciens* and *S. aureus* showed the highest and lowest sensitivity (2 and 8 mg/ml), respectively (Table 3). It is worth noting that the bactericidal activity of the essential oil from Nourabad Mamasani, showing the highest content of thymol and carvacrol, was higher than that of chloramphenicol against both groups of bacteria. Based on these findings, the Nourabad Mamasani population was found to be the best one, offering the conditions allowing plants to produce essential oils with high yield and high content of thymol and carvacrol. This assures a notable antibacterial activity against human pathogens and phytopathogens.

## 3. Materials and Methods

### 3.1. Plant Material

The aerial parts of *O. decumbens* populations were collected from Nourabad Mamasani, Kahnoyeh-Lar, Behbahan, and Dehdasht in Iran. The samples of the plants were harvested during the flowering stage in June 2017. The plant samples were identified by Prof. Ahmad Reza Khosravi and deposited at the herbarium of Biology Department, Shiraz University. The voucher specimens and geographical attributes of *O. decumbens* populations are shown in Table 3.

### 3.2. Essential Oil Isolation

The essential oils from the air-dried flowering aerial parts (50 g for each sample in three replications) were isolated by hydrodistillation using a Clevenger apparatus for 3 h. The collected essential oils were dried over anhydrous sodium sulfate. They were kept at 4 °C and under dark condition until further analysis. 

### 3.3. GC-MS Analysis

The EOs composition was analyzed by gas chromatography-mass spectrometry (GC-MS). GC-MS analyses of the EO samples were carried out using a GC (Model 7890A; Agilent Technologies), equipped with a flame ionization detector (FID) and an HP-5 column (30 m × 0.32 mm i.d.; film thickness 0.25 μm). The program of column thermal started at 60 °C, then increased at the rate of 3 °C/min to 210 °C. Finally, the temperature reached 240 °C at the rate of 20 °C/min and maintained at the final temperature for 8.5 min. The injector temperature was 280 °C. Nitrogen was used as the carrier gas which contained an inlet flow of 1 mL/min. Gas chromatography-mass spectrometry analysis was conducted by the same GC coupled with a mass-spectrometer (Model MS-5975C, Agilent Technologies). The column included a HP-5MS (30 m × 0.32 mm i.d.; film thickness 0.25 μm). The injector and MS detector temperature was 280 °C. The ionization energy was 70 eV. Helium was also used as the carrier gas at 1 mL/min. 

### 3.4. Determination of Minimum Inhibitory Concentration

The antibacterial assay was assessed using Gram-positive bacteria, *Bacillus subtilis*, *Clavibacter michiganensis*, *Curtobacterium flaccumfaciens*, and *Staphylococcus aureus* and Gram-negative bacteria, *Xanthomonas citri*, *Agrobacterium tumefaciens*, and *Escherichia coli*. *Clavibacter michiganensis*, *Curtobacterium flaccumfaciens*, *Xanthomonas citri*, and *Agrobacterium tumefaciens* were supplied by the Department of Plant Protection, Shiraz university, and Bacillus subtilis, *Staphylococcus aureus*, and *Escherichia coli* were obtained from Institute of Biotechnology, Shiraz University. The minimum inhibitory concentration (MIC) of the essential oils was determined by nutrient broth micro-dilution assay [49]. Bacterial species were cultured at 37 °C for 18 h into a nutrient broth medium on a shaker and their density was measured at OD_600_ using a spectrophotometer. The EOs were dissolved in sterile nutrient broth medium containing 5% dimethyl sulfoxide and 2.5% tween 80 and the serial two-fold dilutions of essential oils were prepared within the concentration ranges of 0.0312–16 mg/ml. Chloramphenicol (0.5%, Sina Darou, Iran; ranging from 0.00625 to 5 mg/ml) was used as positive control, while 5% dimethyl sulfoxide and 2.5% tween 80 (as solvent for EOs) were considered as negative control for all the bacteria. An amount of 100 μL of the diluted essential oils was added to each well containing 20 μL fresh bacterial suspension (OD = 0.5 at 600 nm). The microplates were covered and incubated overnight at 37 °C in a shaking incubator to mix the contents of the wells and then 20 μL of 0.5% *p*-iodonitrotetrazolium chloride (INT, Sigma-Aldrich) solution was added and incubated for 30 min in a shaking incubator. The MIC was recorded as the first concentration of the essential oil with no bacterial growth, which prevented the color change of the medium [50]. Before adding INT to the wells, the absorbance of bacterial growth was measured at 600 nm and the results of both methods were checked to obtain the real MIC values. The experiments were repeated twice with three replicates.

### 3.5. Determination of Minimum Bactericidal Concentration

The minimum bactericidal concentration (MBC) of *O. decumbens* essential oils was determined [51]. The amount of 5 μL of MIC and higher concentrations (without INT) were spread on nutrient agar plates and incubated at 37 °C overnight. The first concentration that showed no bacterial growth on nutrient agar medium was considered as MBC. The experiments were repeated twice with three replicates.

### 3.6. Statistical Analysis

The significant differences of the means of experimental groups were determined using one-way ANOVA followed by Duncan’s multiple range test (*P* < 0.05). All data analyses were performed using SAS software version 9.4. Pearson correlation analysis was conducted by SPSS software version 21 (SPSS Inc., Chicago, IL, USA). Biplot chart was drawn by Minitab 16 statistical software. The experiment was carried out in three replicates.

## 4. Conclusions

The results of this work shed light on the positive relationship between thymol and carvacrol contents and the level of antibacterial activity exhibited by the *O. decumbens* essential oils. The present study highlighted also the remarkable variability in the essential oil composition, content, and antibacterial activity of four Iranian populations of *O. decumbens*. These differences might be due to a number of factors including plant genetics and environmental factors characterizing each growing area for *O. decumbens* populations. Notably, the essential oil from the Nourabad Mamasani population exhibited a pronounced antibacterial activity and this was correlated with the highest essential oil contents of thymol and carvacrol. Interestingly, the MBC of this essential oil revealed to be better than that of the positive control chloramphenicol. On this basis, the essential oil of Nourabad Mamasani population is a valuable thymol and carvacrol-rich source which may be considered as a promising natural alternative to conventional antibiotics to be used to combat pathogens impacting human health and agriculture. Further studies are required to determine the effect of genetics and environmental factors on the content of thymol and carvacrol in the field. These measures might be effective in improving the amount of thymol and carvacrol constituents of the plants aimed to exploit them in the agrifood and pharmaceutical industries. The findings of this study could provide effective information for the commercial cultivation and breeding of *O. decumbens* in order to exploit its valuable compounds.

## Figures and Tables

**Table 1:** Antibacterial activity (expressed as minimum inhibitory concentration, MIC (mg/ml)) of different essential oils from *Oliveria decumbens* populations against Gram-positive and Gram-negative bacteria.

	Bacteria	Mean	Essential Oils				Chloramphenicol (mg/ml)
			Nourabad Mamasani	Dehdasht	Behbahan	Kahnoyeh	
Gram-positive	*S. aureus*	0.63 ± 0.25 ^a*^	0.5	0.5	1	0.5	0.2
	*B. subtilis*	0.45 ± 0.41 ^a^	0.0625	0.25	0.5	1	0.8
	*C. michiganensis*	0.44 ± 0.13 ^a^	0.5	0.25	0.5	0.5	0.025
	*C. flaccumfaciens*	0.47 ± 0.39 ^a^	0.125	0.25	0.5	1	0.025
Average MIC against Gram-positive bacteria		0.49	0.30 ± 0.24 ^a^	0.31 ± 0.13 ^a^	0.63 ± 0.25 ^ab^	0.75 ± 0.29 ^b^	0.26 ± 0.37 ^a^
Gram-negative	*X. citri*	1.25 ± 0.5 ^b^	1	1	2	1	0.4
	*A. tumefaciens*	0.44 ± 0.38 ^a^	0.25	0.25	0.25	1	0.025
	*E. coli*	0.69 ± 0.38 ^ab^	0.25	0.5	1	1	0.4
Average MIC against Gram-negative bacteria		0.78	0.5 ± 0.43 ^a^	0.58 ± 0.38 ^a^	1.08 ± 0.88 ^a^	1 ± 0 ^a^	0.28 ± 0.22 ^a^
Average MIC against both groups of bacteria		0.61	0.38 ± 0.32 ^a^	0.43 ± 0.28 ^ab^	0.82 ± 0.59 ^bc^	0.86 ± 0.24 ^c^	0.27 ± 0.29 ^a^

* Means followed by the same letters (a, b, c) are not significantly different according to Duncan’s multiple range test at the significance level *P* < 0.05. The results of significant differences are means ± standard deviations of the populations and bacteria.

**Table 2:** Antibacterial activity (expressed as minimum bactericidal concentration, MBC (mg/ml)) of different essential oils from *Oliveria decumbens* populations against Gram-positive and Gram-negative bacteria.

	Bacteria	Mean	Essential Oils				Chloramphenicol (mg/ml)
			Nourabad Mamasani	Dehdasht	Behbahan	Kahnoyeh	
Gram-positive	*S. aureus*	8 ± 5.66 ^b*^	4	8	4	16	5
	*B.subtilis*	7 ± 2 ^ab^	4	8	8	8	1.6
	*C. michiganensis*	4.25 ± 2.87 ^ab^	1	4	4	8	1.6
	*C. flaccumfaciens*	2 ± 1.41 ^a^	1	1	2	4	3.2
Average MBC against Gram-positive bacteria		5.31	2.5 ± 1.73 ^a^	5.25 ± 3.4 ^ab^	4.5 ± 2.52 ^ab^	9 ± 5.03 ^b^	2.85 ± 1.62 ^a^
Gram-negative	*X. citri*	4.5 ± 2.52 ^a^	2	4	4	8	5
	*A. tumefaciens*	6 ± 6.73 ^a^	2	2	4	16	0.1
	*E. coli*	6 ± 6.73 ^a^	2	2	4	16	5
Average MBC against Gram-negative bacteria		5.5	2 ± 0 ^a^	2.67 ± 1.15 ^a^	4 ± 0 ^a^	13.33 ± 4.62 ^b^	3.37 ± 2.83 ^a^
Average MBC against both groups of bacteria		5.39	2.29 ± 1.25 ^a^	4.14 ± 2.85 ^a^	4.29 ± 1.8 ^a^	10.86 ± 5.01 ^b^	3.07 ± 2.01 ^a^

* Means followed by the same letters (a, b) are not significantly different according to Duncan’s multiple range test at the significance level *P* < 0.05. The results of significant differences are means ± standard deviations of the populations and bacteria.

**Table 3:** Geographical characteristics and voucher specimens of *Oliveria decumbens* populations from which essential oils were obtained.

No.	Population	Province	Latitude (N)	Longitude (E)	Altitude (m)	Voucher Specimen
1	Nourabad Mamasani	Fars	30°08′21.6”	51°33′16.3”	1268	55079
2	Kahnoyeh, Lar	Fars	27°57′59.6”	53°24′11.8”	758	55076
3	Behbahan	Khuzestan	30°32′09.2”	50°23′36.5”	394	55075
4	Dehdasht	Kohgiluyeh and Boyer-Ahmad	30°51′07.3”	50°35′14.5”	971	55080
